# Dynamic changes in nocturnal blood glucose levels are associated with sleep-related features in patients with obstructive sleep apnea

**DOI:** 10.1038/s41598-020-74908-x

**Published:** 2020-10-21

**Authors:** Jung-Ick Byun, Kwang Su Cha, Ji Eun Jun, Tae-Joon Kim, Ki-Young Jung, In-Kyung Jeong, Won Chul Shin

**Affiliations:** 1 Department of Neurology, Kyung Hee University School of Medicine, Kyung Hee University Hospital At Gangdong, 892 Dongnam-ro, Gangdong-gu, Seoul, 134-727 Republic of South Korea; 2 Department of Neurology, Seoul National University Hospital, Seoul National University College of Medicine, Seoul, Republic of South Korea; 3 Department of Endocrinology and Metabolism, Kyung Hee University School of Medicine, Kyung Hee University Hospital At Gangdong, 892 Dongnam-ro, Gangdong-gu, Seoul, 134-727 Republic of South Korea; 4 Department of Neurology, Ajou University School of Medicine, Suwon, Republic of South Korea

**Keywords:** Sleep disorders, Type 2 diabetes

## Abstract

Obstructive sleep apnea (OSA) has a bidirectional relationship with insulin resistance conditions; however, the mechanism remains unclear. This study aimed to compare dynamic nocturnal glucose changes among patients with OSA of varying levels of severity and evaluate temporal changes associated with the cardinal features of OSA (sympathetic hyperactivation, intermittent hypoxemia, and sleep fragmentation) in nondiabetic subjects. Nocturnal glucose was measured with a continuous glucose monitoring device every 5 min during polysomnography (PSG). The OSA features were evaluated using heart rate variability (HRV), minimum saturation, and electroencephalography. Eleven subjects with moderate to severe OSA and 12 subjects with no or mild OSA were evaluated. Those with moderate to severe OSA showed an increasing trend in blood glucose levels after sleep onset, whereas those without or with mild OSA showed a decreasing trend (F = 8.933, *p* < 0.001). Delta band power also showed different trends during sleep between the two groups (F = 2.991, *p* = 0.009), and minimum saturation remained lower in the moderate to severe OSA group than in the no or mild OSA group. High degrees of coupling between nocturnal glucose levels and each OSA feature were observed. Altered trends in nocturnal glucose in moderate to severe OSA may reflect glucose intolerance and result in metabolic consequences. Managing the features of sleep-related OSA may have implications for metabolic management in the future.

## Introduction

Obstructive sleep apnea (OSA) is characterized by recurrent partial or complete upper airway collapse during sleep. It is currently recognized as an important health issue that can affect a variety of organs in the cardiovascular, neurologic, respiratory, and endocrine systems^1^. Up to 40% of patients with OSA have type 2 diabetes mellitus (T2DM)^2^, and OSA is more prevalent in patients with insulin resistance conditions, such as obesity and T2DM, than in the general population^3^. The mean nocturnal glucose level was higher in T2DM patients with OSA than in those without OSA regardless of body mass index (BMI)^4^. These two conditions are known to have bidirectional and independent associations^5,6^. However, the mechanism underlying their relationship remains unclear.

Patients with OSA typically experience intermittent hypoxemia, sleep fragmentation, and sympathetic hyperactivation. Blood glucose is strictly controlled by the neuroendocrine system^7^, and these cardinal features of OSA may explain its metabolic consequences. Intermittent hypoxia and frequent arousal during sleep can increase sympathetic activity and oxidative stress and can cause systemic inflammation and hormonal imbalances, leading to insulin resistance and beta-cell dysfunction^8,9^. However, the evidence of these associations have been derived from limited animal or experimental studies. Moreover, the OSA feature that has the strongest impact on glycemic control remains unknown.

Elucidation of the dynamic relationship between changes in blood glucose levels and OSA features observed in real time during sleep may help explain the physiological relationship between OSA and metabolic dysregulation. One study evaluated dynamic changes in glucose every 20 min during sleep in moderate to severe OSA patients and assessed their relationships with respiratory events, heart rate elevation, and sleep fragmentation^10^. However, the mean HbA1c level of the included patients was 6.4, and approximately a quarter of them had diabetes, which makes it difficult to evaluate the true association between nocturnal glucose changes and the sleep-related features of OSA.

A continuous glucose monitoring (CGM) device allows the measurement of the blood glucose concentration and the monitoring of dynamic glucose changes during sleep^11,12^. A CGM study involving patients with OSA showed higher glycemic variability in the OSA patients than in the controls; glycemic variability may lead to increased organ damage^13^.

Wavelet coherence analysis allows the evaluation of time-varying and frequency-specific coupling between two time series^14^. The fluctuations in the glucose level and OSA features after sleep onset can be decomposed into different frequency components, and the time-varying coherence between the two signals can be calculated at each underlying frequency. Previous studies showed dynamic coupling between CGM data and physical activity^15^ or electroencephalogram (EEG) power^16^ during sleep in patients with type 1 diabetes mellitus using wavelet coherence analysis.

This study aimed to compare dynamic changes in nocturnal glucose levels between nondiabetic individuals with and without moderate to severe OSA using a CGM device during polysomnography (PSG). Moreover, to determine which OSA features were associated with the fluctuations in glucose levels, we performed wavelet coherence analysis between the CGM data and sleep-related OSA features (autonomic nervous system activity, sleep fragmentation, and hypoxia) during sleep.

## Results

### Clinical features and demographics

A total of 27 patients were considered for enrollment; one patient was excluded because of a high HbA1c level (6.9%), one patient was excluded because of morbid obesity (BMI 42.5 kg/m^2^), and two patients were excluded because of poor CGM data. Twelve of the included subjects had no or mild OSA (6 had no OSA, and 6 had mild OSA), and the rest had moderate to severe OSA (5 had moderate OSA, and 6 had severe OSA). Those with moderate to severe OSA had a higher BMI than those without OSA (24.7 ± 2.4 kg/m^2^ vs. 27.7 ± 3.0 kg/m^2^, *p* = 0.036). The PSG results showed a shorter total sleep time, higher arousal index and Apnea–Hypopnea Index (AHI) scores, and lower minimum saturation level in those with moderate to severe OSA than in those with no or mild OSA. There were no significant differences in metabolic parameters or 3-day CGM data between the two groups. Although the number of patients with insulin resistance (homeostasis model assessment of insulin resistance [HOMA-IR] > 2.5) was higher in the moderate to severe OSA group, it was statistically nonsignificant (*p* = 0.131) (Table 1).

**Table 1:** Clinical characteristics of the subjects.

	No or mild OSA	Moderate to severe OSA	*p*-value
	n = 12	n = 11	
Age (years)	42.6 ± 7.1	42.1 ± 12.0	0.422
Sex (male)	9 (75.0)	10 (90.9)	0.315
BMI (kg/m^2^)	24.7 ± 2.4	27.7 ± 3.0	0.036
Neck circumference (cm)	36.4 ± 3.0	38.2 ± 2.4	0.121
Waist circumference (cm)	86.5 ± 7.8	92.4 ± 6.6	0.139
Polysomnography			
TST (min)	318.4 ± 58.2	258.2 ± 68.1	0.014
N1%	16.1 ± 7.4	28.2 ± 21.0	0.116
N2%	45.2 ± 9.6	37.4 ± 10.4	0.085
N3%	23.6 ± 11.2	19.8 ± 12.3	0.479
R%	15.2 ± 5.5	14.6 ± 9.1	0.951
WASO (min)	8.2 ± 3.6	17.3 ± 17.0	0.559
Sleep latency (min)	7.6 ± 5.4	6.5 ± 8.3	0.090
REM latency (min)	90.3 ± 36.8	88.3 ± 51.5	0.895
Sleep efficacy (%)	89.6 ± 3.6	81.1 ± 17.3	0.782
Arousal index (/hr)	26.6 ± 17.9	45.6 ± 17.6	0.010
AHI (/hr)	6.3 ± 4.3	36.7 ± 18.4	< 0.001
Minimum saturation (%)	88.6 ± 3.2	76.0 ± 8.6	< 0.001
Metabolic measures			
Fasting glucose (mg/dl)	98.8 ± 11.1	102.2 ± 7.1	0.267
HbA1c (%)	5.2 ± 0.4	5.4 ± 0.4	0.336
Insulin (µU/mL)	5.9 ± 2.4	8.2 ± 4.7	0.356
HOMA-IR	1.48 ± 0.70	2.07 ± 1.17	0.356
HOMA-IR > 2.5	1 (8.3)	4 (36.4)	0.131
FFA (µEq/L)	495.4 ± 180.1	428.6 ± 248.7	0.310
Cholesterol (mg/dl)	205.5 ± 22.3	207.5 ± 28.3	0.666
TG (mg/dl)	140.9 ± 74.7	169.5 ± 138.3	0.712
HDL (mg/dl)	50.7 ± 12.0	57.0 ± 10.3	0.131
LDL (mg/dl)	140.4 ± 23.3	133.0 ± 32.9	0.518
3-Day CGM measures			
Average	101.3 ± 29.5	111.0 ± 9.8	0.517
SD	17.2 ± 5.3	17.3 ± 3.8	0.665
MAD%	9.6 ± 5.1	10.6 ± 5.6	0.644

OSA, obstructive sleep apnea; BMI, body mass index; TST, total sleep time; WASO, wake after sleep onset; AHI, apnea–hypopnea index; HOMA-IR, homeostatic model assessment for insulin resistance; FFA, free fatty acid; TG, triglyceride; HDL, high-density lipoprotein; LDL, low-density lipoprotein; CGM, continuous glucose monitoring; SD, standard deviation; MAD, mean amplitude.

### Dynamic changes in blood glucose levels during sleep

**Figure 1:**
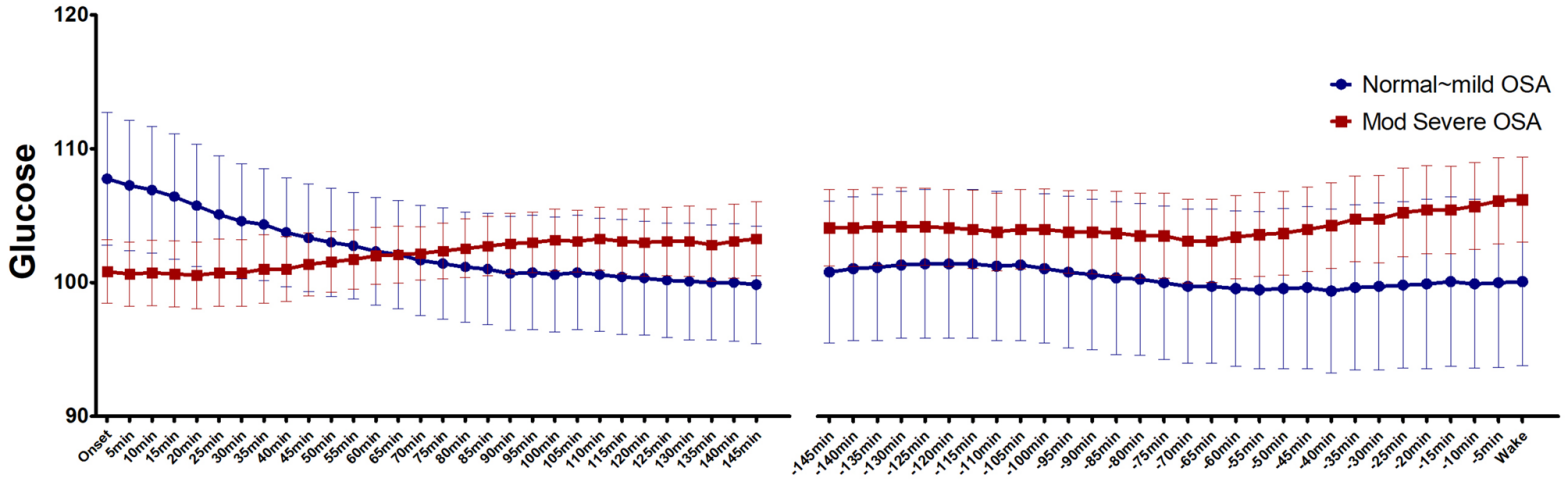
Dynamic changes in nocturnal glucose between no or mild OSA and moderate to severe OSA patients. Glucose was measured every 5 min with a continuous glucose monitoring device. Left: first part of sleep (from sleep onset to 145 min after sleep onset), Right: second part of sleep (from 145 min before waking to waking). Abbreviation: OSA, obstructive sleep apnea.

We analyzed nocturnal glucose levels during the period between sleep onset and awakening. The glucose level remained within the normoglycemic range regardless of OSA severity, and no significant difference between the two groups was found at any time point. There was a significant time by group interaction (F = 8.933, *p* < 0.001) during the first half of the period. Those with normal or mild OSA had a decreasing trend in the glucose level after sleep onset, whereas those with moderate to severe OSA had an increasing trend. The interaction was not statistically significant during the second half of the period (Table 2, Fig. 1).

**Table 2:** Mean area of significant coherence and significant coherence level.

Fluctuation range		DFA1 (α1)	DFA2 (α2)	Min Sat	Delta power	Alpha power	Theta power
Range 1 (10–30 min)	Significant area	1081.0 ± 976.8	1062.7 ± 1067.1	666.0 ± 762.4	969.7 ± 750.4	866.7 ± 819.4	934.0 ± 708.1
	Significant coherence	0.77 ± 0.17	0.77 ± 0.17	0.67 ± 0.32	0.75 ± 0.24	0.74 ± 0.24	0.81 ± 0.03
Range 2 (30–90 min)	Significant area	601.9 ± 1003.0	463.3 ± 1060.4	829.5 ± 1030	952.2 ± 1362.2	469.3 ± 749.8	913.0 ± 1530.4
	Significant coherence	0.45 ± 0.40*	0.32 ± 0.41*	0.60 ± 0.36	0.57 ± 0.38	0.32 ± 0.41*	0.50 ± 0.41*
Range 3 (90-160 min)	Significant area	83.5 ± 185*#	70.7 ± 181.3*	112.4 ± 251*#	96.1 ± 190*#	66.5 ± 128.3*	81.6 ± 188*#
	Significant coherence	0.27 ± 0.41*	0.23 ± 0.39*	0.26 ± 0.40	0.34 ± 0.43	0.22 ± 0.39*	0.23 ± 0.40*

Significant difference compared to Range 1 * (*p* < 0.01).

Significant difference compared to Range 2 # (*p* < 0.01).

Significant area, area of significant coherence; Significant coherence, mean significant coherence value; Range 1, fluctuation period range 10–30 min; Range 2, fluctuation period range 30–90 min; Range 3, fluctuation period range 90–1600 min.

Abbreviations: DFA, detrended fluctuation analysis; Min Sat, minimum saturation.

### Dynamic changes in sleep-related OSA features during sleep

There was a significant group by time interaction with regard to the delta power (F = 2.991, *p* = 0.009). There was an increase in the delta power after sleep onset in the no or mild OSA group; however, no such change was evident in the moderate to severe OSA group. A significant time effect was observed for the delta (F = 4.035, *p* = 0.001) and theta powers (F = 5.456, *p* < 0.001) during the first half of the period and for the alpha power (F = 4.669, *p* = 0.007) during the second half of the period. A significant group effect was observed for only minimum saturation during both the first and second halves of the period (F = 24.516, *p* < 0.001 and F = 9.335, *p* = 0.006, respectively). No significant effect of time, group or interaction was found for HRV (Supplementary Fig. S1 online).

### Coherence between glucose- and sleep-related OSA features during sleep

There was no significant difference between the sleep-related features with regard to either the significant coherence value or its area in any fluctuation period range. Additionally, no effects of group (no or mild OSA vs. moderate to severe OSA groups) were significant. The significant coherence value and its area were also similar between those with moderate to severe OSA and those with no or mild OSA (see Supplementary Table S2 online). The coherence value between the sleep-related OSA features was more significant than that with the blood glucose level (Table 2, Supplementary Fig S2 online).

Repeated measures analysis of variance (RMANOVA) showed significant effects of range on the significant coherence value and its area for all sleep-related features. The significant coherence value was greater for faster oscillation (Range 1) than for slower oscillation (Range 2 or Range 3) for all sleep-related features, except for minimum saturation and delta power. The mean area of significant coherence was greater for faster oscillation (Range 1) than slower oscillation (Range 3) for all of the features. Except for the DFA2 and alpha power, Range 2 was also greater than Range 3 (see Supplementary Table S1 online).

## Discussion

A difference in the trends in nocturnal blood glucose level was evident between the moderate to severe OSA and no or mild OSA groups. Those with no or mild OSA had a decreasing trend in glucose levels during the first period of sleep, whereas an increasing trend was seen in those with moderate to severe OSA. The difference in trends was also significant for delta power, and the minimum saturation level was lower in those with moderate and severe OSA than in those with no or mild OSA. High degrees of time-varying and frequency-specific coupling were evident between nocturnal glucose fluctuations and all of the sleep-related OSA features, especially for faster oscillation. These features may cause distinct dynamic changes in nocturnal glucose levels in subjects with moderate to severe OSA, eventually resulting in metabolic syndrome.

People with no or mild OSA had a decreasing trend in glucose levels after sleep onset, especially during the first period. This result was in line with a previous CGM study involving healthy subjects that showed a decrease in nocturnal glucose levels predominantly during rapid eye movement (REM) sleep^12^. Prolonged fasting during the daytime results in a significant decrease in glucose levels, even without physical activity; however, glucose remains constant or decreases only minimally during sleep^17^. A parallel decrease in glucose production and its utilization was suggested to be the reason for the constant nocturnal glucose level^18^. During sleep, the brain accounts for a significant portion of whole-body glucose consumption^19^, and its activity is reduced by 30–40%. Although we did not analyze glucose changes according to sleep stage, a previous study suggested that an increase in brain activity during REM sleep may be the reason for the decline in glucose levels during sleep^12^. Insulin clearance increases by 40% during the first half of sleep, which may also decrease nocturnal glucose utilization^20^.

On the other hand, those with moderate to severe OSA had an increasing trend in nocturnal glucose levels after sleep onset. In a previous study, the decreasing trend in glucose levels during REM sleep was reversed during apneic events in patients with mild or moderate OSA^21^. Glucose levels remained constant during the nonrapid eye movement (NREM) period with or without sleep-disordered breathing events^21^, and nocturnal glucose levels may have an overall increasing trend in OSA patients, as observed in our study. A dynamic increase in the nocturnal plasma glucose level was also reported in patients with moderate to severe OSA (AHI ≥ 20/hr) in association with sympathetic and adrenocortical activation^10^. The association requires careful interpretation because the study included patients with diabetes and morbid obesity. We excluded those with diabetes or morbid obesity to evaluate the true relationship between the nocturnal glucose level and sleep-related features.

Altered nocturnal glucose variations in moderate to severe OSA patients may be due to changes in sleep-related OSA features, including arousal and oxygen desaturation^6^. The trend in changes in delta band power differed between the two groups during the first period of sleep. One could simply assume that a reduced delta power in OSA patients may increase cerebral glucose consumption and reduce nocturnal glucose levels. However, glucose regulation is not merely dependent on cerebral glucose consumption. The suppression of slow-wave sleep in healthy adults can lead to a decrease in insulin sensitivity, which leads to impaired glucose tolerance^22^. Moreover, even brief arousals can lead to surges in sympathetic activity^23^. Hypoxia that persists during sleep is also known to decrease insulin sensitivity by inducing sympathetic hyperactivity^24^ and to increase the hepatic glucose output^21^. Desaturation in patients with moderate to severe OSA was reported to have a temporal association with a surge in nocturnal glucose^25^. An increasing trend in nocturnal glucose may serve as an early marker for insulin resistance, even before changes in conventional parameters such as HOMA-IR. Differences in autonomic activity during sleep between the two groups were not evident in this study. Although we used poincaré plots with artifact correction, artifacts from arousal and sleep-disordered breathing may have negated the difference.

Coherence analysis showed that glucose fluctuations during sleep were coupled with sleep-related OSA features, including minimum saturation, HRV and EEG band power. Our significant coherence value for glucose and EEG power was comparable to the result from a previous study of patients with type 1 diabetes^16^. No significant differences were observed between the sleep-related features. Because all of the OSA features are highly interconnected, they may have similar associations with glucose levels. There was no significant effect of OSA group, which is in line with the result from a previous study that suggested that overnight glucose was affected by sleep-related features, not by the severity of the OSA itself^10^. As in a previous study^16^, coherence between glucose/sleep-related factors, including EEG power, was higher for faster oscillation (Range 1) than slower oscillation (Range 2 or 3). This is in line with the results of a study that showed that rapid changes in nocturnal glucose were associated with frequent awakening from sleep in pediatric type 1 diabetic patients^26^.

The findings of this study should be interpreted in the context of its limitations. This was a single-center study with a small number of subjects, and most of them were male, which makes it difficult to generalize the results. However, we strictly excluded those with diabetes and morbid obesity. Nevertheless, the mean BMI was higher in the moderate to severe OSA group than in the no or mild OSA group, which may have influenced the difference in nocturnal glucose changes. Although none of the participants had circadian rhythm disorder, sleep onset and wake times varied among the subjects, which also may have affected glucose metabolism. This study simply demonstrated associations between trends in glucose levels and sleep-related factors, not causal relationships. Moreover, the physiological and clinical implications of frequency-specific coupling between the glucose level and sleep-related factors should be further investigated.

Moderate to severe OSA was associated with an increasing trend in glucose levels after sleep onset; this relationship was associated with sleep-related features, such as sleep fragmentation, desaturation and autonomic dysfunction. Distinct changes in nocturnal glucose levels in patients with moderate to severe OSA may eventually result in metabolic syndrome. Understanding the mechanistic basis for the time-varying association between glucose levels and OSA features during sleep may have future implications for the management of metabolic consequences. Future crossover studies with a larger number of patients with and without positive airway pressure treatment may reveal a causal relationship between the two.

## Methods

### Subjects

This was a prospective observational single-center study performed in Kyung Hee University Hospital at Gangdong. Patients who underwent overnight polysomnography (PSG) due to the clinical suspicion of OSA were considered for enrollment. Age- and sex-matched healthy volunteers without sleep disturbances from the same region also participated in this study and were included in the normal group. All participants underwent CGM monitoring during the PSG. Individuals with obesity (BMI ≥ 35 kg/m^2^), diabetes (HbA1c ≥ 6.5% or a fasting glucose level ≥ 126 mg/dL as defined by the Korean Diabetes Association guidelines^27^), cardiac disease (e.g., angina pectoris, myocardial infarction, or atrial fibrillation), or other sleep disorders (e.g., REM sleep behavior disorder, narcolepsy, circadian rhythm disorder, or restless legs syndrome) were excluded. Patients with poor sleep efficiency (sleep efficiency < 50%) or poor-quality CGM data were also excluded from this study.

This study was carried out in accordance with the principles of the Declaration of Helsinki and approved by the Institutional Review Board of Kyung Hee University Hospital at Gangdong (IRB No.: 2016–08-020). Informed consent to participate was obtained from the enrolled patients and healthy volunteers.

### PSG

PSG was performed using a digital polygraph system (Grass-Telefactor twin version 2.6, West Warwick, RI, USA) according to standard protocols. The data were manually scored according to the *American Academy of Sleep Medicine (AASM) Manual for the Scoring of Sleep and Associated Events*, version 2.4^28^. The AHI was calculated as the mean number of apnea and hypopnea events per hour of sleep. OSA severity was categorized according to commonly used cutoffs: no OSA (AHI < 5/hr), mild OSA (5/hr ≤ AHI < 15/hr), moderate OSA (15/hr ≤ AHI < 30/hr), and severe OSA (AHI ≥ 30/hr). The participants consumed regular meals and fasted for at least 2 h before and during the PSG. Taking medications that could affect sleep, consuming caffeine, engaging in excessive physical activity and smoking were discouraged during the study period.

### Glucose data: CGM

A CGM device (iPro2, Medtronic, Northridge, CA, USA) placed in the abdominal subcutaneous tissue was used to measure blood glucose levels every 5 min. The device converts the raw signal of the interstitial glucose concentration into an estimate of the blood glucose concentration by a calibration process that was performed with self-monitored blood glucose levels every 12 h^29^. Subjects were instructed in the use of the device. Subjects spent three days with the CGM: two nights with normal daily activities outside the laboratory and the last night with in-laboratory overnight PSG. The average, highest and lowest blood glucose levels, standard deviation (SD), and mean amplitude (MAD) were measured. Glucose levels during PSG were matched every five minutes after sleep onset with PSG data.

Blood samples were collected to determine metabolic profiles, including the levels of insulin, glucose and lipids, after PSG. HOMA-IR was calculated to evaluate insulin resistance, and those with HOMA-IR > 2.5 were defined as having insulin resistance^30^.

### Sleep-related OSA features (HRV, EEG, and minimum saturation data)

#### HRV

Overnight lead II ECG data (sampling rate 400 Hz) were extracted from routine PSG data and converted into consecutive R-R intervals for the HRV analysis using Kubios Premium, version 3.0.2^31^. Detrended fluctuation analysis (DFA), which measures correlations within the data over different time scales, was consecutively measured every five minutes after sleep onset: DFA1 (α1) and DFA2 (α2) were obtained from plots by default within the ranges of 4–16 beats and 16–64 beats, respectively. DFA1 and DFA2 have been reported to be relatively less affected by artifacts, which makes them appropriate for use in OSA patients^32^.

#### Minimum saturation

The minimum saturation level every five minutes during sleep was recorded and matched with the CGM data.

#### EEG power

Six EEG channels were analyzed: two frontal (F4/M1 and Fz/M2), two central (C4/M1 and Cz/M2), and two occipital (O2/M1 and Oz/M2) channels. The EEG data (sampling rate 500 Hz) were bandpass filtered (0.5–70 Hz) and analyzed using MATLAB 2017b software. The EEG data were downsampled to 200 Hz, and a bandpass filter with zero phase shift was applied in the range of 0.5–50 Hz to reduce background noise. The data were then segmented into 4-s segments, and segments with voltages exceeding the range of − 200 to 200 µV were rejected. To observe spectral characteristics during sleep, the power spectra of each 5-min epoch were calculated using Welch’s method, with nonequispaced fast Fourier transform (NFFT) = 512, 50% overlapping window, and 4-s window segments, and matched to the CGM data. The frequency bands were defined as follows: delta (0.5–4 Hz), theta (4–8 Hz), and alpha (8–12 Hz) bands.

These 5-min averaged HRV (DFA1 and DFA2), EEG power (delta, theta, and alpha band power), and minimum saturation data were aligned simultaneously with the glucose data from the CGM device.

### Wavelet coherence analysis

Overnight (from sleep onset to wake) coupling between changes in nocturnal glucose levels and sleep-related OSA features was analyzed using wavelet coherence analysis, as in a previous study^16^. The wavelet coherence toolbox provided by Grinsted in MATLAB 2014b^33^ was used to compute time-varying coupling or coherence between two signals that underwent wavelet decomposition. This allowed us to extract coherence values every 5 min for each of 144 underlying oscillation scales ranging from 10 to 160 min. As in the previous study^16^, we used three empirically derived ranges in the fluctuation period [Range 1 (10- to 30-min fluctuation period), Range 2 (30- to 90-min fluctuation period), and Range 3 (90- to 160-min fluctuation period)] and analyzed only the coherence values outside of the “cone of influence”. Statistically significant coherence values (*p* < 0.05) were identified by Monte Carlo simulations (N = 500). We calculated the average significant coherence values and areas with significant coherence.

### Statistical analysis

Data were compared between the no or mild OSA (AHI < 15/h) and moderate or severe OSA (AHI ≥ 15/h) groups. Continuous data were compared using the nonparametric Mann–Whitney U test, and categorical variables were compared using the chi-square test.

Differences in changes in dynamic glucose and sleep-related features during sleep were compared between the two groups using RMANOVA with within-subject factors (time, every 5 min consecutively) and between-subject factors (group). Mauchly’s test for sphericity was performed, and when the assumption of sphericity was violated, a Greenhouse–Geisser correction was applied. Because the average duration from sleep onset to awakening was 336 min, and the minimum duration was 293.5 min, we divided the total sleep period into two periods and analyzed each period separately: the first period (every 5 min from sleep onset to 145 min after sleep onset) and the second period (every 5 min from 145 min before awakening to the wake time).

The results of the coherence analysis were compared according to the sleep-related features and OSA groups and according to the fluctuation period ranges using RMANOVA for within-subject factors (sleep-related features or fluctuation period ranges) and between-subject factors (groups) with a post hoc Wilcoxon signed-rank test.

The level of significance was set at *p* < 0.05, and the significance for the post hoc test was set at *p* < 0.01. All statistical comparisons were performed with SPSS (Version 22.0, Chicago, IL, USA).

## Supplementary information

Supplementary Information 1.

Supplementary Information 2.

Supplementary Information 3.

## Data Availability

Continuous glucose monitoring and polysomnographic signals and preprocessed data analyzed during the current study are not publicly available due to the need to comply with privacy regulations. Summary statistics are available from the corresponding author upon reasonable request.
