# Supplementary figures and images for: Dynamic changes in nocturnal blood glucose levels are associated with sleep-related features in patients with obstructive sleep apnea
Source: Sci Rep. 2020 Oct 21;10:17877. doi: 10.1038/s41598-020-74908-x (PMC7578637; doi:10.1038/s41598-020-74908-x)

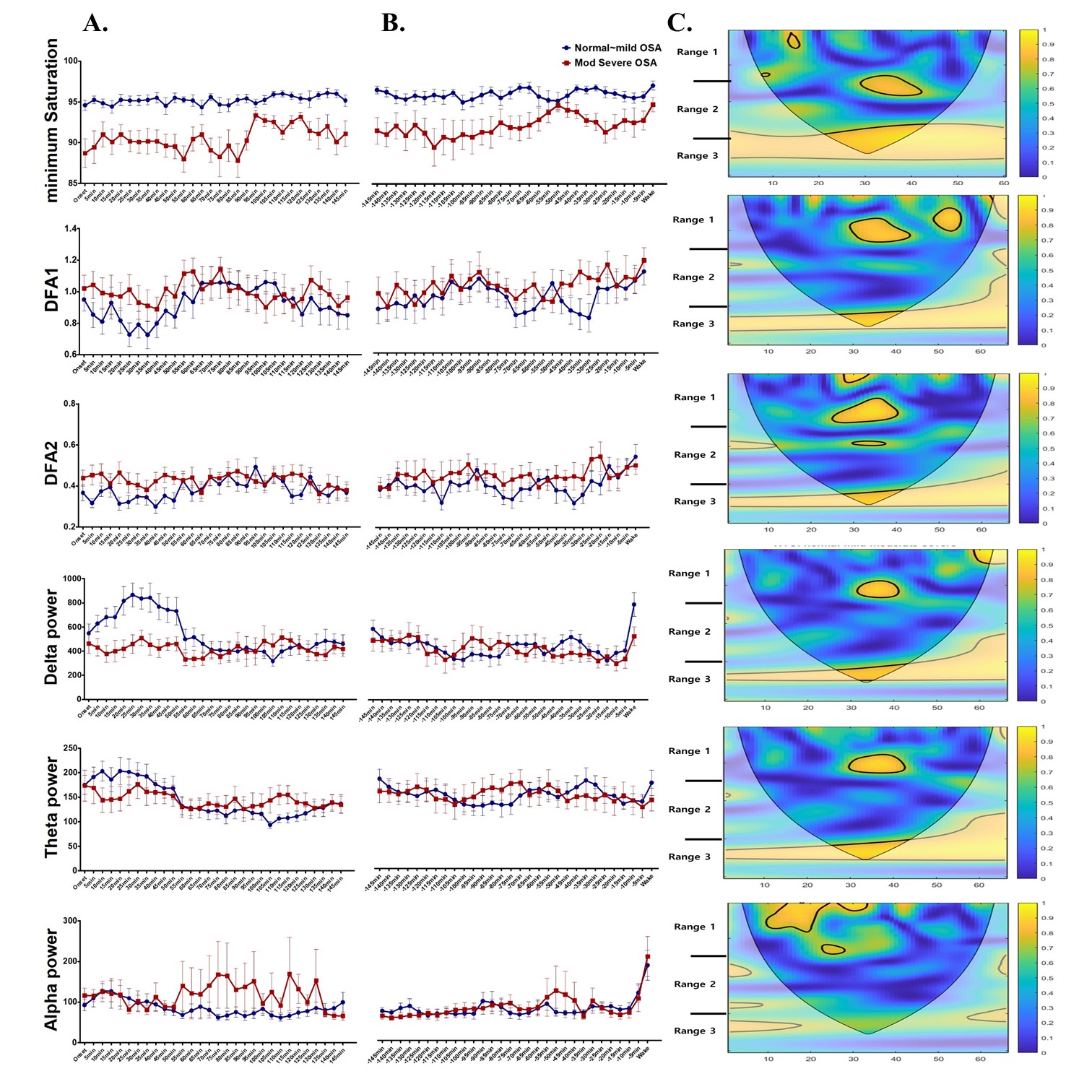

Supplement: Supplementary file 1 — Supplementary Information 1. [file 41598_2020_74908_MOESM1_ESM.jpg]

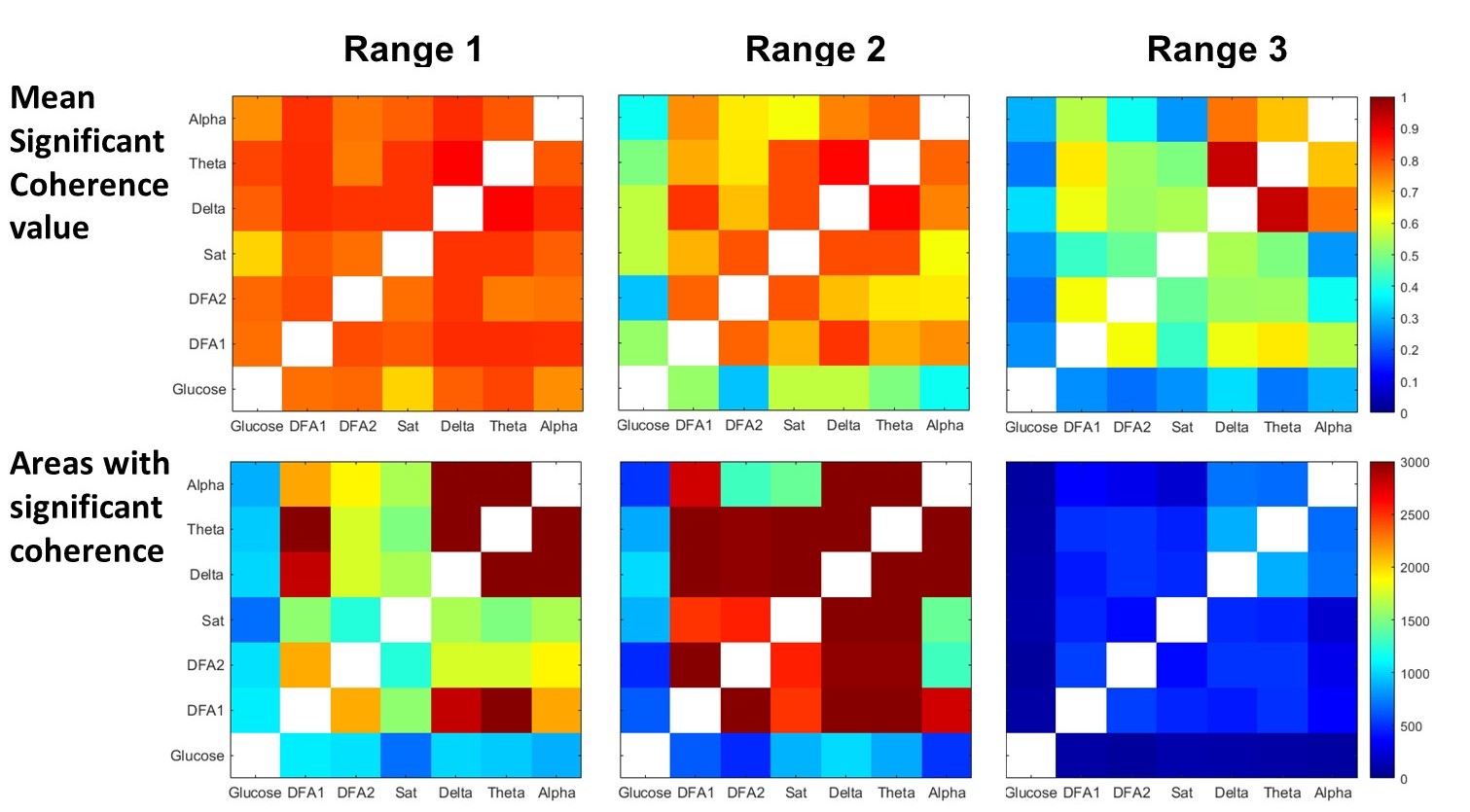

Supplement: Supplementary file 2 — Supplementary Information 2. [file 41598_2020_74908_MOESM2_ESM.jpg]
