# Supplementary material for: Dynamic changes in nocturnal blood glucose levels are associated with sleep-related features in patients with obstructive sleep apnea
Source: Sci Rep. 2020 Oct 21;10:17877. doi: 10.1038/s41598-020-74908-x (PMC7578637; doi:10.1038/s41598-020-74908-x)
Supplement: Supplementary file 3 — Supplementary Information 3. [file 41598_2020_74908_MOESM3_ESM.doc]

**Dynamic changes in nocturnal blood glucose levels are associated with sleep-related features in patients with obstructive sleep apnea**

Jung-Ick Byun, MD, PhD^a^; Kwang Su Cha^b^; Ji Eun Jun MD, PhD^c^; Tae-Joon Kim^d^; Ki-Young Jung, MD, PhD ^b^; In Kyung Jeong MD, PhD^c*^; Won Chul Shin MD, PhD^a*^

^a^Department of Neurology, Kyunghee Hee University Hospital at Gangdong, Kyung Hee University School of Medicine, Seoul, South Korea.

^b^Department of Neurology, Seoul National University Hospital, Seoul National University College of Medicine, Seoul, South Korea

^c^Department of Endocrinology and Metabolism, Kyung Hee University Hospital at Gangdong, Kyung Hee University School of Medicine, Seoul, South Korea

^d^Department of Neurology, Ajou University School of Medicine, Suwon, Republic of South Korea

*These authors contributed equally to this work.

**Supplementary Fig S1: Dynamic changes in sleep related features between patients with no or mild OSA and those with moderate or severe OSA and its coherence with nocturnal glucose levels.**

Sleep-related features measured every 5 minutes (minimum saturation recorded, heart-rate variability (DFA1, DFA2) and EEG power averaged every consecutive 5 minutes) during A: the first part of sleep (from sleep onset to 145 min after sleep onset), B: the second part of sleep (from 145 min before waking to waking), C: Heat map demonstrating frequency-specific and time-varying coherence between each sleep-related feature and glucose over the entire sleep period in one patient (54-year-old female) without OSA. Yellow areas bounded by a black line indicate the areas of statistically significant coherence (p<0.05) based on Monte Carlo simulations. Range 1: 10-30 min; Range 2: 30-90 min; Range 3: 90-160 min. The lighter shaded area indicates the cone of influence (COI).

Abbreviations: DFA, detrended fluctuation analysis; OSA, obstructive sleep apnea

**Supplementary Fig S2: Coherence matrix of significant coherence values and areas of the significant coherence between the nocturnal glucose levels and sleep-related OSA features.**

Abbreviations: DFA, detrended fluctuation analysis; Sat, minimum saturation; OSA, obstructive sleep apnea

Supplementary Table S1. Results of repeated measure ANOVA (Within subject factor: Range)

|  |  | Time | Time x Group | Group |  |
| --- | --- | --- | --- | --- | --- |
| Sig coherence | DFA1 (α1) | F=12.742,  p<0.001 | ns | ns | Range 1 > 2, Range 1 > 3 |
|  | DFA2 (α2) | F=18.286,  p<0.001 | ns | ns | Range 1 > 2, Range 1 > 3 |
|  | Min sat | F=9.400,  p=0.001 | ns | ns |  |
|  | Delta power | F=6.815,  p=0.004 | ns | ns |  |
|  | Alpha power | F=14.669,  p<0.001 | ns | ns | Range 1 > 2, Range 1 > 3 |
|  | Theta power | F=20.410  p<0.001 | ns | ns | Range 1 > 2, Range 1 > 3 |
|  |  |  |  |  |  |
| Sig area | DFA1 (α1) | F=9.984  p<0.001 | ns | ns | Range 1 > 3, Range 2 > 3 |
|  | DFA2 (α2) | F=8.282  p=0.002 | ns | ns | Range 1 > 3 |
|  | Min Sat | F=9.162  p<0.001 | ns | ns | Range 1 > 3, Range 2 > 3 |
|  | Delta power | F=7.251  p=0.007 | ns | ns | Range 1 > 3, Range 2 > 3 |
|  | Alpha power | F=8.881  p=0.002 | F=4.416  p=0.027 | ns | Range 1 > 3 |
|  | Theta power | F=6.244  p=0.013 | ns | ns | Range 1 > 3. Range 2 > 3 |

Range 1, fluctuation period range 10-30 minutes; Range 2, fluctuation period range 30-90 minutes; Range 3, fluctuation period range 90-160 minutes

Abbreviations: Sig area, area of significant coherence; Sig coherence, mean significant coherence value; DFA, detrended fluctuation analysis; Min sat, minimum saturation.

Supplementary Table S2: Difference in significance coherence values and areas between patients with moderate to severe OSA and those with no or mild OSA.

|  |  |  | No or mild OSA | Moderate to  severe OSA | p-value |
| --- | --- | --- | --- | --- | --- |
|  |  |  | n=12 | n=11 |  |
| Significant  coherence  value | Range 1 | Min sat | 0.67±0.32 | 0.67±0.33 | 0.459 |
|  |  | DFA1 | 0.81±0.03 | 0.73±0.25 | 0.712 |
|  |  | DFA2 | 0.74±0.24 | 0.81±0.04 | 0.854 |
|  |  | Delta power | 0.83±0.03 | 0.67±0.33 | 0.268 |
|  |  | Theta power | 0.80±0.04 | 0.82±0.02 | 0.157 |
|  |  | Alpha power | 0.81±0.03 | 0.67±0.33 | 0.498 |
|  | Range 2 | Min sat | 0.66±0.31 | 0.53±0.42 | 0.664 |
|  |  | DFA1 | 0.33±0.41 | 0.58±0.37 | 0.21 |
|  |  | DFA2 | 0.47±0.42 | 0.15±0.34 | 0.1 |
|  |  | Delta power | 0.48±0.42 | 0.67±0.33 | 0.533 |
|  |  | Theta power | 0.41±0.43 | 0.60±0.39 | 0.253 |
|  |  | Alpha power | 0.20±0.37 | 0.45±0.43 | 0.152 |
|  | Range 3 | Min sat | 0.14±0.33 | 0.39±0.45 | 0.113 |
|  |  | DFA1 | 0.30±0.44 | 0.23±0.40 | 0.597 |
|  |  | DFA2 | 0.21±0.39 | 0.25±0.42 | 0.75 |
|  |  | Delta power | 0.29±0.42 | 0.40±0.46 | 0.529 |
|  |  | Theta power | 0.14±0.34 | 0.33±0.46 | 0.232 |
|  |  | Alpha power | 0.14±0.34 | 0.31±0.43 | 0.3 |
| Significant  area | Range 1 | Min sat | 933.5±913.1 | 374.3±426.8 | 0.096 |
|  |  | DFA1 | 1159.6±890.0 | 995.4±1101.1 | 0.498 |
|  |  | DFA2 | 923.6±1148.9 | 1214.4±1002.3 | 0.242 |
|  |  | Delta power | 772.5±435.5 | 1184.8±965.7 | 0.325 |
|  |  | Theta power | 793.3±584.2 | 1087.6±823.4 | 0.389 |
|  |  | Alpha power | 1093.8±808.9 | 618.8±792.5 | 0.065 |
|  | Range 2 | Min sat | 893.9±1041.7 | 759.3±1063.3 | 0.535 |
|  |  | DFA1 | 459.8±1044.3 | 756.9±981.4 | 0.131 |
|  |  | DFA2 | 581.6±1250.1 | 334.2±848.2 | 0.133 |
|  |  | Delta power | 1159.4±1718.9 | 726.2±851.3 | 0.851 |
|  |  | Theta power | 926.6±1976.7 | 898.1±924.4 | 0.281 |
|  |  | Alpha power | 164.9±349.9 | 801.4±932.6 | 0.087 |
|  | Range 3 | Min sat | 140.3±337.2 | 82.1±109.0 | 0.29 |
|  |  | DFA1 | 129.4±241.1 | 33.4±78.6 | 0.546 |
|  |  | DFA2 | 81.1±216.3 | 59.5±143.5 | 0.968 |
|  |  | Delta power | 95.3±220.8 | 96.9±161.8 | 0.625 |
|  |  | Theta power | 68.3±216.2 | 96.0±162.5 | 0.339 |
|  |  | Alpha power | 21.4±56.3 | 115.6±166.2 | 0.175 |

Range 1, fluctuation period range 10-30 minutes; Range 2, fluctuation period range 30-90 minutes; Range 3, fluctuation period range 90-160 minutes

Abbreviations: DFA, detrended fluctuation analysis; Min sat, minimum saturation.
